# Implementing non-invasive prenatal testing for aneuploidy in a national healthcare system: global challenges and national solutions

**DOI:** 10.1186/s12913-017-2618-0

**Published:** 2017-09-19

**Authors:** Rachèl V. van Schendel, Carla G. van El, Eva Pajkrt, Lidewij Henneman, Martina C. Cornel

**Affiliations:** 1 Department of Clinical Genetics, Section of Community Genetics, Amsterdam Public Health research institute, VU University Medical Center, P.O. Box 7057, 1007 MB Amsterdam, The Netherlands; 2 Department of Obstetrics and Gynecology, Fetal Medicine Unit, Academic Medical Centre, Amsterdam, The Netherlands

**Keywords:** Down syndrome, Prenatal screening, Non-invasive prenatal testing, Implementation, Qualitative analysis, Innovation

## Abstract

**Background:**

Since the introduction of non-invasive prenatal testing (NIPT) in 2011, mainly by commercial companies, a growing demand for NIPT from the public and healthcare professionals has been putting pressure on the healthcare systems of various countries. This study identifies the challenges of establishing a responsible implementation of NIPT for aneuploidy in prenatal healthcare, by looking at the Netherlands.

**Methods:**

A mixed methods approach involving 13 stakeholder interviews, document analysis and (participatory) observations of the Dutch NIPT Consortium meetings were used. The Diffusion of Innovation Theory and a Network of Actors model were used to interpret the findings.

**Results:**

Implementation of NIPT was facilitated by several factors. The set-up of a national NIPT Consortium enabled discussion and collaboration between stakeholders. Moreover, it led to the plan to offer NIPT through a nationwide research setting (TRIDENT studies), which created a learning phase for careful implementation. The Dutch legal context was perceived as a delaying factor, but eventually gave room for the parties involved to organise themselves and their practices.

**Conclusions:**

This study shows that implementing advanced technologies with profound effects on prenatal care benefit from a learning phase that allows time to carefully evaluate the technical performance and women’s experiences and to enable public debate. Such a coordinated learning phase, involving all stakeholders, will stimulate the process of responsible and sustainable implementation.

## Background

Prenatal care is a dynamic and continuously evolving field. New technologies are introduced to improve care for mother and child and to offer parents reproductive options. Implementing a new technique in a pre-existing setting is often a multilevel process involving different stakeholders and causes changes in organisation, policy and finance [1]. In this article, the challenges and dynamics of implementing non-invasive prenatal testing (NIPT) for foetal aneuploidy in a national prenatal healthcare setting will be discussed.

NIPT is a revolutionary technique for testing for Down syndrome (trisomy 21) and other foetal aneuploidies using cell-free DNA (cfDNA) in maternal blood [2]. NIPT can be performed as early as 9-week gestation [3], has a high sensitivity (>99% for Down syndrome), and a false positive rate of less than 0.1% [4]. These test characteristics make NIPT a suitable screening test for foetal anomalies, leading to a significant reduction in women referred for invasive testing and thus lowering the number of procedure-related miscarriages [5, 6].

NIPT made its first commercial debut in the United States in 2011. NIPT’s great potential and advantages have made it a highly requested test, now offered in over 60 countries [7, 8], with prices rapidly decreasing. However, variability in test prices between countries limits adoption of NIPT and creates inequality of access [9]. Moreover, quality control should extend to the non-laboratory aspects like education of professionals, adequate information provision, and development of counselling guidelines [10]. Since NIPT causes such a change in the landscape of prenatal screening, it presents a global challenge of offering adequate, equal and equitable services for all women seeking prenatal testing.

In the Netherlands, for reasons that will be discussed in this article, it was considered expedient to incorporate NIPT into the national healthcare system in which Down syndrome screening is coordinated by a National Institute of Public Health. Since April 2014, NIPT has been offered as part of a nationwide evaluation study to pregnant women who are at an elevated risk of carrying a foetus with Down syndrome (or trisomy 18 or 13) based on the first-trimester combined test (FCT) (risk cut-off 1:200) or medical history [11], and from April 2017 to all pregnant women irrespective of their risk (TRIDENT studies, see Table 1). The Netherlands is one of the first countries worldwide to implement NIPT in public prenatal care. This was established within a few years. Here we provide more insight into the process of implementing NIPT by presenting the constraining and enabling factors and the conditions for successful implementation. We performed document analysis, (participatory) observations and qualitative interviews with key stakeholders. Two theoretical frameworks were used: the Diffusion of Innovation Theory and a Network of Actors model. This case study can serve to inform future implementation of NIPT in other countries or the implementation of other novel technologies in prenatal care.

**Table 1:** Short description of the TRIDENT studies on the implementation of NIPT in The Netherlands

	TRIDENT studies (Trial by Dutch laboratories for Evaluation of Non-Invasive Prenatal Testing (NIPT))
Background & study period	In the Netherlands, NIPT became available in April 1, 2014 as part of the first TRIDENT study for pregnant women with an indication for invasive diagnostic testing based on an increased risk for aneuploidy (e.g. Down syndrome)(risk ≥1:200) at first trimester combined screening. In addition, women with a medical history, e.g. previous child with Down syndrome can have direct access to NIPT. NIPT is offered after counselling to women attending one of the eight specialized Prenatal Diagnosis Units [11]. A two-year license, based on the Population Screening Act, was obtained from the Minister of Health, and later extended for another 2 years (2018). As of April 2017, NIPT will be offered to all pregnant women irrespective of risk (TRIDENT-2 study). A three-year license for this study was obtained.
NIPT analyses	NIPT is performed by clinical genetic laboratories of the Dutch University Medical Centres, using (in-house validated) massively parallel shotgun sequencing.
Dutch NIPT Consortium	The TRIDENT studies were designed and proposed by the national multidisciplinary NIPT Consortium, formed in 2011. The NIPT Consortium is membered by all stakeholders involved in prenatal care (including obstetricians, clinical geneticists, midwives, laboratory specialists, the Dutch Genetic Alliance, ethicists, and researchers).
Study aim	To investigate all relevant aspects of the implementation of NIPT in the Dutch prenatal screening program. The studies will evaluate two parts: Part I. Organization, logistics, test-performance, costs [11]. Part II. Women’s decision-making, uptake, preferences, psychosocial aspects [35].
Funding and cost	The laboratory tests for high-risk women were funded in 2014 by the Ministry of Health, but since 2015 covered by the insurance companies (women can be charged for their yearly compulsory own risk excess (385 euros)). As of 2017, women will pay 175 euros for NIPT as a first-tier screening test. The study evaluations are funded by The Netherlands Organisation for Health Research and Development (ZonMw, grants no. 200340002 (TRIDENT-1) and 543002001 (TRIDENT-2).

### The pre-existing down syndrome screening regime in the Netherlands

To understand the context in which NIPT implementation took place we first briefly describe Down syndrome screening in the Netherlands. The Netherlands has a well-organised prenatal care system. However, the introduction of prenatal screening for foetal abnormalities, such as neural tube defects and Down syndrome, has been a lengthy process accompanied by numerous discussions [12]. The idea of prenatal screening for all pregnant women raised concerns, both in Parliament and wider society, regarding social pressure to screen and ‘collective eugenics’ [12]. For Down syndrome screening, the limited test characteristics of the first-trimester combined test (FCT) posed a problem [12]. From January 1st 2007 onwards all pregnant women are asked whether they wish to be informed about prenatal screening for Down syndrome and, if so, they are then given the choice for FCT. From 2007 until 2015, the costs of FCT (~150 euro) were reimbursed to women with elevated risk (≥36 years or medical history), who could also opt to proceed directly for invasive testing. However, since 2015, maternal age alone is no longer considered sufficient indication for invasive diagnostic testing (i.e. no longer a high-risk group), and costs for FCT are only reimbursed to women with a medical history. The uptake of the FCT is approximately 27% [13], which is relatively low compared to other European countries [14, 15].

In Dutch prenatal care, multiple stakeholders are involved. Midwives play an important role as the majority of pregnant women (~85%) begin their prenatal care in a midwifery practice [16, 17]. With an elevated risk (≥1:200) at FCT, the woman is referred to a foetal medicine unit. Facilities for invasive diagnostic aneuploidy tests are available in the eight university medical centres and their satellites [18]. The Dutch National Institute for Public Health and the Environment-Centre for Population Screening (RIVM-CvB) coordinates and monitors prenatal screening and develops standardised information.

Prenatal screening is regulated by the Dutch Population Screening Act, established in 1996 to protect people against potentially harmful screening. A license must be obtained before organising some forms of screening, such as population screening for disorders with “no available treatment” or prevention, including prenatal screening for Down syndrome [12].

## Methods

### Design

A qualitative study design was used that involved semi-structured interviews, document analysis and (participatory) observations). The Medical Ethical Committee of the VU University Medical Center Amsterdam approved the study protocol. Two theoretical frameworks were used to guide the interviews and to interpret and present our findings: the Diffusion of Innovation Theory and a Network of Actors model.

### The diffusion of innovation theory

**Fig. 1:**
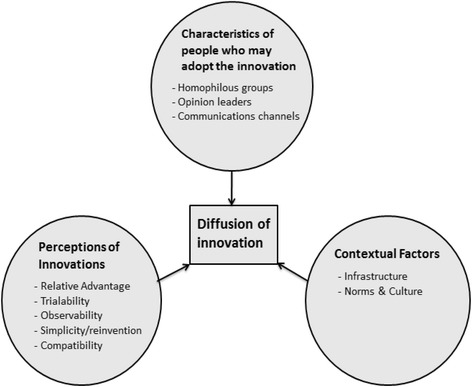
The clusters of critical dynamics described by the Diffusion of Innovation Theory, based on: [1, 19]

Diffusion is the process by which an innovation is adopted over time among the members of a system. Cain & Mittman describe ten critical dynamics that govern how fast new medical technologies are diffused in a healthcare setting [1]. These dynamics can be grouped into three clusters, as described below (Fig. 1) [19].

#### Perceptions of the innovation

Five dynamics are included in this cluster [19]. ‘Relative advantage’ means that the more benefit is anticipated from an innovation, the more likely it will get adopted. ‘Trialability’ refers to the possibility to try out an innovation on a smaller scale first. ‘Observability’ is whether potential users can witness others try out the innovation. ‘Simplicity/reinvention’ concerns whether innovations can be altered or simplified by users, leading to a faster adoption. ‘Compatibility’ is the ability to coexist with social patterns and technologies already in place and the values and needs of the potential adopters [1, 19, 20].

#### Characteristics of the people who adopt the innovation

The characteristics of the members of the group adopting an innovation will also affect the ease of diffusion. Innovations tend to spread faster among ‘homophilous groups’: groups with similar characteristics and interests. ‘Opinion leaders’ are key actors in the adoption of the innovation whose power and (social) media exposure influences others [1]. ‘Communication channels’ are the paths through which people communicate about the innovation [1, 20].

#### Contextual factors

Diffusion is influenced by contextual factors, such as the presence of existing norms and culture, and infrastructure [1]. For an innovation to succeed, other systems or technologies should already be in place.

### Network of actors model

**Fig. 2:**
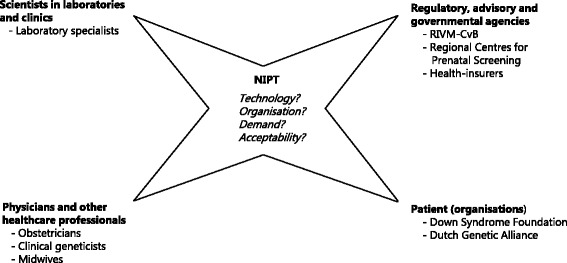
Interviewees categorized by the four different stakeholder groups in a Network of Actors, based on [21]

To broaden the analysis as proposed by Cain & Mittman and to better understand the interaction between various organisations and professionals involved, we used a Network of Actors model previously described by Achterbergh et al. (see Fig. 2) [21]. A technological innovation usually starts in a niche. To enable scaling up, the existing regime of practices, rules and routines needs to be adapted. Stakeholders from different fields in science and healthcare, patients and public, as well as regulatory bodies, need to enter a process of mutual learning and attunement [22].

### Semi-structured interviews

The Network of Actors model served to identify the relevant stakeholder groups (Fig. 2). Semi-structured interviews were conducted with 15 key informants (four laboratory specialists, two gynaecologists, two clinical geneticists, two midwives, two patient organisation representatives (Down Syndrome Foundation and the Dutch Genetic Alliance), one health insurance advisor and two representatives from a Dutch screening organisation) between April and July 2013. The interviews were conducted at the workplace of the interviewee, except for two telephone interviews. The outcomes of the interviews were presented at a meeting (September 2014) of the Dutch NIPT Consortium (interactive workgroup of all institutions, organisations and stakeholders involved with NIPT) as feedback for the implementation process and to validate the research findings.

#### Interview guide

The interview protocol was based on the theoretical frameworks and the document analysis and covered the following topics: stakeholders’ opinions on the current FCT screening versus NIPT, thoughts on the impact and uptake of NIPT in the Netherlands, views on the constraining and enabling factors in the implementation of NIPT, opinions about the collaboration between stakeholders in the implementation process and views on adaptations and changes needed for successful implementation.

### Document analysis and (participatory) observations

To better understand the implementation process and serve as a basis for the interviews, relevant documents were collected and analysed including reports of the Health Council, documents of the Dutch national NIPT Consortium (i.e. emails, reports, minutes, etc.), and newspaper articles. The meetings of the NIPT Consortium were attended and observed.

### Data analysis

The interviews were audiotaped and transcribed verbatim. Content analysis was performed using the qualitative software programme ATLAS.ti 5.2. Transcripts were read and discussed by three researchers (RvS, LH, CvE) and codes were created for recurring topics in the text. For the document analysis, relevant documents (emails, newspaper articles, letters, minutes) were content analysed and coded (RvS, CvE). The theoretical frameworks were used to cluster the codes of the interviews and document analysis into main themes.

## Results

Based on the interviews and document analysis, three phases in the implementation process of NIPT into the Dutch public healthcare were identified. These phases and the constraining and enabling factors relevant for the implementation of NIPT will be discussed below.

### Phase I: Innovation and its advocates in science and healthcare

#### Observability & Relative advantage

Stakeholders in Dutch science quickly became aware of the advantages of NIPT in terms of its high detection rate, low false positive rate, safety and potential for innovative research. Dutch medical centre laboratories had been conducting research on non-invasive tests for several years. The laboratories collaborated with leading international research groups and one of the first publications about cfDNA to detect trisomy 21 by massively parallel sequencing had three Dutch co-authors [23]. These studies enabled local ‘niches’ to become acquainted with the technology and allowed for ‘observability’ for Dutch laboratories and healthcare professionals. Also, an alternative use of NIPT, Rhesus D (RhD) typing in RhD negative mothers, was studied [24], proven viable and introduced into the Dutch screening programme in 2011.

Healthcare professionals, most notably obstetricians, were soon convinced of the advantages of NIPT compared to conventional foetal aneuploidy tests. The reduction of miscarriage risk was a great relief for obstetricians who experienced women suffering an iatrogenic miscarriage after invasive prenatal diagnosis.

*“This morning I spoke to a colleague who had a patient with ruptured membranes after an invasive test. Those things are very dramatic… a child dies that was wanted and healthy. So, if there is a new [non-invasive] test that is very good […] I am very motivated to help get that implemented.” (obstetrician #01)*

#### Opinion leaders

Certain key actors stood out in getting NIPT implemented. They contacted the national press and raised awareness among pregnant women, healthcare professionals and policymakers. These ‘opinion leaders’ put NIPT on the agenda and raised expectations. For instance, in March 2011 a prominent researcher stated in the Dutch media that NIPT could be implemented by the end of that year [25].

#### Simple but complex

From a technical stance NIPT is a highly sophisticated and complex technology demanding a costly high-tech infrastructure and trained lab staff. However, for healthcare workers, the simplicity and superiority of the test strongly favoured diffusion. The midwives and obstetricians, although not having had actual experience with NIPT yet, perceived the test as relatively simple because only a blood draw is required and it is easy to explain to pregnant women, in particular when compared to the FCT.

*“I think it [procedure and counselling of NIPT] will be much simpler for us […] we notice that the counselling for the current prenatal screening [FCT] is rather complicated.” (midwife #01)*

### Phase II: Increasing demand and offer

#### Communication channels

Starting March 2011, there has been abundant coverage of NIPT-related news in the Dutch media. Moreover, pregnant women shared information and experiences on pregnancy forums. Consequently, many pregnant women soon became aware of the existence of a new, safer test. In the media, questions arose as to why this test was not yet available in the Netherlands. When the public became aware that NIPT was available abroad, women started asking for their samples to be sent abroad or went to Belgium or Germany themselves to undergo NIPT in a clinic collaborating with commercial companies. The public demand can be seen as an important driving force in the implementation of NIPT in the Netherlands.

*“The public is going to strongly demand it [NIPT], and we have already seen some examples of that in the newspaper; like ‘why is this test [NIPT] not available yet’? The Minister has to explain ‘well that’s not allowed because’…well they have to come up with a really good excuse then, because public opinion is very important in politics.” (obstetrician #01)*

In 2012, obstetricians supporting NIPT also started sending their patients’ samples abroad. However, at the beginning of 2013 the Ministry of Health forbade obstetricians doing so as no governmental license had been issued [26], the relevance of which we will discuss below.

#### Commercial offer

With the increase of tests performed abroad, the Dutch prenatal diagnostics centres witnessed significant declines in the number of invasive procedures for aneuploidies. In laboratories and among obstetricians, uncertainties were expressed regarding the quality of tests performed abroad and it was clear that equity of access was at stake. Laboratory specialists and healthcare professionals feared they were lagging behind in their service development.

*“It is less clear how it [NIPT] is done there, and you don’t want companies to start offering it [NIPT] with another sensitivity or specificity. We are talking about a prenatal test here, which has a lot of implications.” (laboratory specialist #01)*

*“You notice that, for university medical centres, it [commercial NIPT offer abroad] is a stimulus to also go along with this development. They don’t want to fall behind, so in that sense it puts a certain pressure on the system.” (screening organisation representative #01)*

### Phase III: Changing practice, culture and structure: Towards a new regime of prenatal screening

#### Cooperation

Until 2011, different medical centres were each validating procedures for NIPT. Although important, according to some stakeholders this has also led to a somewhat ‘aimless’ start with little communication and cooperation between different clinics.

*“…you saw all kinds of local initiatives, this group was doing this, the other that, people were working independently of each other.” (clinical geneticist #01)*

In 2011, lab specialists from four clinical genetics centres discussed setting up a validation study on the feasibility and real-time detection rate of NIPT. Additionally, several obstetrician and clinical geneticists met regularly in a standing advisory committee of the Prenatal Screening Section of the RIVM-CvB. They monitored the developments in NIPT closely and some committee members decided to organise a national consortium in which all stakeholders participated. Several meetings followed to design a research proposal for a national validation study of stored samples [27]. However, this so-called NITRO study (Non-Invasive Trisomy Research) proposal was turned down due to financial reasons and because there was no intention to report back test-results to patients. Nevertheless, the meetings of the Consortium created a basis for collaboration that later led to an implementation study (Table 1).

#### Norms and culture

As described earlier, the introduction of prenatal Down screening in the Netherlands in 2007 was accompanied by (ethical) debate and controversy. In contrast, nation-wide implementation of NIPT for foetal RhD typing occurred without any public or political debate, as its only aim is to safeguard foetal health. Implementing NIPT for aneuploidies was more ethically complex, but since many parties wanted to avoid the delays witnessed with introducing FCT, a more rapid implementation was pursued. Stakeholders mentioned that in their view, society had already accepted population screening for Down syndrome, so, they argued, NIPT could be regarded as simply a better and safer alternative.

*“Technically speaking, NIPT is just a test. The framework for implementing that [Down syndrome screening] has already been created with the combined test. It is about autonomous reproductive choices and informed choice.” (screening organisation representative #01)*

However, especially some Christian political parties were sceptical towards NIPT since they feared it would lead to the normalization of abortions for Down syndrome [28]. According to some stakeholders, these issues might have encouraged some politicians to stir up debate and try to delay the implementation of NIPT.

*“Yes, the topic is just not so convenient for politics, because there’s always an abortion bit attached to it. They [politicians] just don’t feel like doing it [discussing the implementation of NIPT], it produces hassle, so when they have the chance to delay it, they will.” (obstetrician #01)*

Also, in newspapers and on web forums, some individuals and organisations expressed their concerns and opposition to NIPT. It was debated whether Down syndrome should be screened for at all [29].

#### Legal context

Professionals initially assumed that NIPT, as a better test, could simply replace current screening tests. However, it soon became apparent that if any element of the screening trajectory was to be replaced, a request for a new license was required by the Population Screening Act. Since stakeholders had already teamed up for the NITRO study proposal in 2012, it was relatively easy to continue the collaboration in the Consortium to request a license for all eight university medical centres in the Netherlands.

**Fig. 3:**
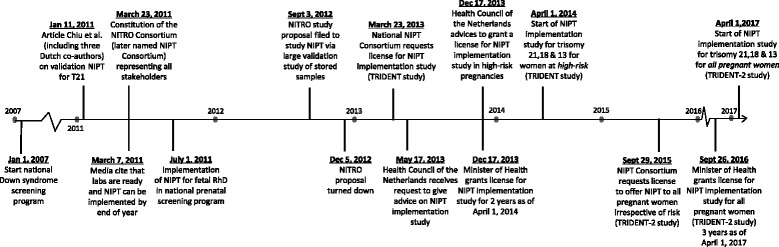
Timeline of relevant events in the implementation process of NIPT in Dutch public prenatal care

Many stakeholders at the time criticised the Population Screening Act and mentioned that the process of obtaining the license delayed the implementation. Writing the proposal took many months and, once it had been finalised in March 2013, the Health Council, an advisory institution of the government, needed several more months to evaluate this document (Fig. 3).

*“Yes, on a technical level we are all ready for it. We can start tomorrow. The only thing that is now hindering the implementation of NIPT in the Netherlands is the Population Screening Act license.” (clinical geneticist #01)*

Even though all stakeholders agreed that obtaining the license was a constraining factor, some mentioned that this process also ensured that stakeholders were in agreement on the implementation strategy and everyone was sufficiently trained and the information material was ready.

*“Well look, it gets delayed because the license is required. […] This is an advantage in the sense that it [implementation of NIPT] is now being done very carefully.” (patient organisation representative #01)*

#### Trialability

The key element of the license proposal was an implementation study to gather relevant data on real-time test performance and the experience and preferences of women. Even though, in theory, it benefits the adoption of an innovation to start on a small scale (*trialability*), a study limited to just one centre would have been irresponsible with NIPT since many women already wanted to have the test. Moreover, most of the eight clinical genetic laboratories wanted to offer NIPT since they had the know-how and necessary equipment and did not want to lag behind. Therefore, a first nationwide evaluation research study was planned (TRIDENT study, see Table 1). Based on the international findings at that time [30, 31], the scenario chosen for the first TRIDENT study was FCT as the primary screening test, and offer women with increased FCT test result (risk ≥1:200) or maternal age ≥ 36 years the choice between NIPT, invasive testing or no follow-up test. The Health Council was supportive, but advised against offering NIPT on the basis of maternal age alone, as this was not considered to be a good indicator for aneuploidy risk [32]. In cooperation with ‘Erfocentrum’, the Dutch national genetic resource and information centre, a website was launched in Dutch (www.meeroverNIPT.nl) to provide information about the study and its inclusion criteria and to address questions about NIPT raised by the public [33].

#### Compatibility

The TRIDENT study could use the population-based screening structure already in place. As explained earlier, prenatal screening is coordinated and monitored by the RIVM-CvB, which distributed adapted counselling- and educational materials. Moreover, the nationwide foundations for prenatal screening organised the postgraduate training of midwives and other healthcare professionals. The existence of these structures helped prepare the implementation of NIPT in an organised and coordinated manner.

“*The RIVM organises the whole screening thing. So the pathways, the whole structure, is already in place. So if you say ‘from this date on we are going to do it’ [offer NIPT], and everybody has been trained, etc., then it should be easy to roll out.” (obstetrician #02*)

However, the fact that a screening system based on the FCT had been implemented shortly before, initially caused some resistance from people who had made huge efforts to implement the FCT.

*“But the people [laboratories] that are doing the combined test, they almost feel like it [implementation of NIPT] is kind of a personal insult. In the end they are really cooperative, so it’s not a reproach that they aren’t cooperating.[…] But I think that I see it more as a risk, a kind of obstacle.” (laboratory specialist #02)*

#### Infrastructure

At the time, the choice to offer NIPT as a contingent screening test in the TRIDENT study was based on the good test performance of NIPT for women at an elevated risk for foetal aneuploidies, and a lack of sufficient data on performance in low-risk pregnancies. By leaving the existing regime of prenatal screening based on the FCT virtually intact, the scenario of an implementation study enabled the Dutch healthcare system to prudently use scarce resources. In addition, the field could learn from the first study experiences, and prepare for a further upscaling if NIPT were to be officially implemented for a larger target group.

For the time being, the study setting resolved uncertainties on how to implement NIPT in prenatal care. Furthermore, the TRIDENT study forced different professional groups to start working together. This was important as the shifting distribution of tasks was a potential source of tension. For instance, NIPT is performed in clinical genetic laboratories while FCT is performed in screening laboratories.

*“Well, you do see, but that’s my personal opinion, a tribal war going on. You observe a flow where the cyto-geneticists see part of their work disappear and are afraid of the future.[…] You see the same happening with the obstetricians in the sense that some think ‘our job is going to be downgraded to sampling blood that will be analysed somewhere else’.” (laboratory specialist #03)*

Eventually, through regular meetings, the national Consortium and TRIDENT study created better cooperation, thereby stimulating mutual understanding and close collaboration between stakeholders.

Lastly, the funding of the TRIDENT study proved a challenge. The Ministry of Health would only start reviewing the license application if funding for the study had been arranged.

Moreover, the Ministry placed great importance on reimbursement by healthcare insurance for NIPT. However, the problem arose that legal decisions concerning healthcare insurance coverage only become effective from the 1st of January of each year.

*“So then you go and talk to the National Healthcare Institute, and you know those trajectories… before you have an appointment with them another six months has passed.” (clinical geneticist #01)*

After the Minister granted a license in December 2013, the TRIDENT study for high-risk women started on April 1, 2014. For that first year, alternative funding had to be found. Eventually, the government decided to fund NIPT until the end of 2014, followed by coverage from healthcare insurance starting from January 2015.

#### TRIDENT-2 study: All pregnant women

Based on the results of large clinical trials [6, 34], in 2015 the NIPT Consortium requested another license to start offering NIPT to all pregnant women irrespective of their risk. After a positive advice from the Health Council, the Minister of Health granted a 3-year license to offer NIPT to all pregnant women as of April 2017 within the context of the TRIDENT-2 study (see Table 1). Women are still able to choose for NIPT as a contingent screening test after FCT, but now will also have the option to have NIPT as their first screening test. A first-tier NIPT costs 175 euro, which is almost similar to the costs for the FCT (~168 euros).

## Discussion

Dutch researchers and healthcare professionals quickly realized the advantages of NIPT in terms of its performance characteristics and safety. Opinion leaders put NIPT on the agenda to implement this test in the Netherlands. Commercial companies started offering NIPT in neighbouring countries, which attracted Dutch pregnant couples. This led to a growing pressure on the healthcare system. A national NIPT consortium enabled closer collaboration between stakeholders and plans to offer NIPT in a research setting with all university medical centres. However, this required a license according to the Population Screening Act which delayed the process of implementation but also gave room for the parties involved to organise themselves and their practices. Once the study was accepted, NIPT was offered in a research setting for high-risk pregnant women and as of April 2017 to all pregnant women. On the one hand, pregnant women can be offered the test, while on the other hand there is time to evaluate and fine-tune the offer of NIPT. Thus, in the Netherlands a coordinated learning phase was established in which screening was combined with research. It allowed stakeholders, such as healthcare professionals, laboratory specialists and policy makers, to attune the organisation, finance and design of NIPT-based screening in public healthcare.

Allyse et al. [8] describe that NIPT should comply with existing legal and ethical frameworks surrounding reproductive technologies in the country where NIPT is to be implemented. In the Netherlands, the possibility to partly use the existing framework of prenatal screening and refer to accepted ethical frameworks for Down screening facilitated the implementation of NIPT. Minear et al. [9] also state that, when implementing NIPT, national discussions are needed to integrate the perspectives of different stakeholders. In the Netherlands, the national NIPT Consortium indeed facilitated discussion between stakeholders and responsible implementation. Mutual understanding was important since one of the challenges was a shift in responsibilities and work content among stakeholders.

Another facilitator of the implementation of NIPT in the Netherlands was the scale of a small country with almost 17 million inhabitants served by eight university medical centres enabling intense communication. Regional Centres for Prenatal Screening and a national coordinating screening organisation allowed for nationwide training of health professionals to update their knowledge and counselling skills.

The offer of NIPT in the Netherlands forms part of a research study [11, 35]. The experiences are evaluated and used to improve the implementation of NIPT in healthcare. New challenges will arise, now NIPT will be offered to all pregnant women as of April 2017. An upscaling of facilities is needed, implying further challenges to laboratory facilities and reimbursement. In addition, questions concerning the ethical framework of prenatal screening will surface anew as NIPT technology might detect more disorders as well as pregnancy-related abnormalities that may be treated to improve the health of mother and child [8, 36]. Furthermore, concerns about routinisation of prenatal screening call for optimising procedures of informed choice [10].

Also in the UK the evaluation of the implementation of NIPT in the national health service (NHS) was organized through a large implementation study (RAPID study) involving eight different hospitals across the UK [37, 38]. This led to the recommendation to fund NIPT in the UK starting in 2017. Other initiatives are seen in Canada (PEGASUS trial) [39] and Germany (currently evaluating NIPT with a decision expected in 2019) [40].

## Conclusion

Perhaps the most important lesson from the Dutch example, but also from the other initiatives (e.g. UK, Canada), is that implementing advanced technologies with such profound effects on prenatal care benefit from a learning phase, that allows time to carefully evaluate the technical performance and women’s experiences and enable public debate about the impact of the implementation. In countries without a national health service or with less strict governmental policies circumstances would perhaps be less favourable, though other mechanisms may apply, such as learning from local initiatives and copying successful service provision. Establishing some form of a coordinated learning phase will positively stimulate the process of responsible and sustainable implementation.

## Abbreviations

cfDNA: cell - free DNA
DNA: Deoxyribonucleic acid
FCT: First - trimester combined test
NHS: National Health Service
NIPT: Non-invasive prenatal testing
NITRO: Non-Invasive TRisomy Research
RAPID study: Reliable, Accurate Prenatal non-Invasive Diagnosis study
RhD: Rhesus D
RIVM-CvB: Dutch National Institute for Public Health and the Environment-Centre for Population Screening
RNA: Ribonucleic acid
T21/18/13: Trisomy 21, 18, 13
TRIDENT study: Trial by Dutch laboratories for Evaluation of Non-Invasive Prenatal Testing
UK: United Kingdom
